# Neutrophil extracellular traps as immunofibrotic mediators in RA-ILD; pilot evaluation of the nintedanib therapy

**DOI:** 10.3389/fimmu.2024.1480594

**Published:** 2024-10-23

**Authors:** Aliki I. Venetsanopoulou, Maria Ntinopoulou, Eleni Papagianni, Nikolaos Koletsos, Paraskevi V. Voulgari, Akrivi Chrysanthopoulou

**Affiliations:** ^1^ Department of Rheumatology, School of Health Sciences, Faculty of Medicine, University of Ioannina, Ioannina, Greece; ^2^ Laboratory of Molecular Immunology, Department of Biological Applications and Technology, School of Health Sciences, University of Ioannina, Ioannina, Greece

**Keywords:** rheumatoid arthritis-interstitial lung disease, neutrophil extracellular traps, interleukin-17A, tissue factor, terminal complement complex, fibroblasts, nintedanib

## Abstract

**Objective:**

Rheumatoid arthritis-associated interstitial lung disease (RA-ILD) is a significant pulmonary complication of RA. This study tried to elucidate the mechanisms enhancing inflammation and causing lung injury in RA-ILD, focusing on the role of neutrophil extracellular traps (NETs). The study also investigated the potential benefits of nintedanib in advanced disease.

**Methods:**

Nine RA-ILD patients and nine healthy controls were included in the study. Inflammatory markers in patients’ circulation were evaluated with immunoassays. The formation of NETs was examined using a citrullinated histone H3 (CitH3) ELISA and cell immunofluorescence. Inflammatory proteins expressed in neutrophils/NETs were studied with real-time qPCR and NET ELISA. To assess the effect of nintedanib, an intracellular tyrosine kinase inhibitor with antifibrotic properties, in RA-ILD a paired study was conducted in five patients before treatment administration and 16 weeks later.

**Results:**

The soluble terminal complement complex sC5b-9 and the levels of CitH3 were significantly elevated in patients with RA-ILD, compared to healthy controls. In addition, neutrophils isolated from RA-ILD patients released NETs enriched with tissue factor and interleukin-17A. Inflammatory NETs had a dynamic role, increasing the fibrotic potential of human pulmonary fibroblasts (HPFs). On the other hand, nintedanib treatment decreased NETs and sC5b-9 levels in RA-ILD patients.

**Conclusion:**

The findings propose an interplay between circulating NETs and HPFs, establishing the immunofibrotic aspects of RA-ILD. They also support the effectiveness of nintedanib in reducing key pathological processes of the disease. Further research is needed to fully understand these mechanisms and optimize treatment strategies for RA-ILD.

## 1 Introduction

Rheumatoid arthritis (RA) is a chronic inflammatory disease that affects the synovium of the joints, leading to joint destruction and bone damage (1). It is more common in women, with an estimated prevalence of 0.5-1% of adults globally (1–3). Besides synovial joints, RA can affect extra-articular sites such as the skin and the lungs, representing a systemic disorder (1, 2, 4, 5).

One of the most prevalent and severe extra-articular manifestations of RA is rheumatoid arthritis-associated interstitial lung disease (RA-ILD) (5). ILD is detected in up to 60% of RA patients via high-resolution computed tomography (HRCT), yet clinically significant in only about 10% of cases (6). The development of RA-ILD is shaped by inflammatory events, induced by autoantibodies and pro-inflammatory cytokines (7, 8). The perpetuation of inflammation can affect the alveolar and interstitial compartments of the lung, resulting in tissue remodeling (9). Fibrosis can cause varying patterns of lung involvement, with usual interstitial pneumonia (UIP) and nonspecific interstitial pneumonia (NSIP) being the most common histopathological features (8). Recently, specific antifibrotic therapies, such as nintedanib and pirfenidone, have been introduced into clinical practice (10–13) and their efficacy is under investigation.

The immune system plays a significant role in RA-ILD progress. Particularly, neutrophils are highly active immune cells that can recruited to tissues in the presence of inflammatory signals, such as immune complexes and complement components, and secrete pro-inflammatory proteins (14–16). Besides inflammation, they can enhance extracellular matrix (ECM) remodeling and lung tissue damaging, through reactive oxygen species (ROS) and proteolytic enzyme production (17–19). In fibrotic lung, a mechanism that can potently perpetuate interstitial inflammation and fibrosis is the release of inflammatory neutrophil extracellular traps (NETs) (20, 21). NETs can closely interact with fibroblasts, reshaping their biology and giving them antigen-presenting cell capabilities and immunofibrotic aspects (21–23).

Moreover, interleukin-17A (IL-17A) is considered a crucial cytokine in the development of RA-ILD by promoting lung tissue remodeling and fibrosis. It stimulates lung fibroblasts, leading to the production of ECM proteins and the differentiation of fibroblasts into myofibroblasts (24). Studies have also shown that IL-17A expression on NETs promotes mesenchymal stem cell differentiation in other autoimmune diseases (19, 25).

In view of the above evidence, considerable uncertainty persists in the precise role of neutrophils in RA-ILD pathogenesis. Therefore, this study aimed to investigate the protein profile of NETs in RA-ILD and their contribution in the immunofibrotic manifestations of the disease. Additionally, the therapeutic impact of nintedanib, a medication approved for fibrotic lung diseases, was also evaluated in patients with RA-ILD.

## 2 Patients and methods

### 2.1 Patients

Patients, included in the study (n=9, **Table 1**), were diagnosed with RA based on the 2010 ACR/EULAR criteria and were over 18 years old. All patients were diagnosed with RA-ILD, as confirmed by clinical examination, CT scans, and pulmonary function tests. UIP was the predominant ILD pattern observed. Despite their lung conditions, none of the patients exhibited active arthritis or other extra-articular manifestations. Moreover, five of the nine patients received the antifibrotic agent nintedanib and were assessed before and after 16 weeks of treatment. Additionally, 9 age- and sex-matched healthy individuals served as controls. Neutrophils, sera, and plasma were isolated from patients with RA-ILD and healthy individuals for analysis. *In vitro* stimulation experiments were conducted on human pulmonary fibroblasts (HPFs). The study protocol was approved by the Ethics Review Board of the University Hospital of Ioannina (Ethics Review Board protocol number:12916,31/5/22), and all subjects provided written informed consent before participating in the study.

**Table 1:** Demographical data and treatment of the nine patients with RA-ILD.

Patient ID	Age/Sex	RA duration (y)	ILD duration (y)	RF/ACPA	Dyspnea	FVC,% (predicted)	DLCO,% (predicted)	ILD Pattern	ESR	CRP	Steroids/dosage (mg)	Treatment	Other extra-articular	Active arthritis
**1.**	86/F	2	2	+/+	yes	108	96	UIP	23	10	5	nintedanib	no	no
**2.**	63/M	5	2	+/+	yes	93	40	UIP-nodules	88	12	7,5	RTX, nintedanib, HCQ	no	no
**3.**	69/F	6	3	+/+	yes	58	41	NSIP	17	5	5	RTX, HCQ, nintedanib	no	no
**4.**	61/F	12	9	+/+	yes	60	33	UIP	39	6	5	RTX, HCQ, nintedanib	no	no
**5.**	72/M	2	2	+/+	yes	98	48	UIP	20	2	5	HCQ, nintedanib	no	no
**6.**	60/M	8	2	+/+	no	90	64	UIP	5	4	0	RTX	no	no
**7.**	48/M	14	4	+/+	no	98	62	UIP	12	6	5	RTX, MTX	no	no
**8.**	72/F	2	2	-/+	yes	79	53	UIP	6	2	5	HCQ, MMF	no	no
**9.**	67/F	4	4	+/-	yes	94	58	UIP	20	2	0	RTX	no	no

ACPA, Anti-citrullinated protein antibodies; HCQ, Hydroxychloroquine; MTX, Methotrexate; RA, Rheumatoid arthritis; RF, Rheumatoid factor; RTX, rituximab;UIP, usual interstitial pneumonia.

### 2.2 Serum and plasma collection

To isolate serum, venous blood was collected in appropriate blood collection tubes (BD Vacutainer^®^ SST II Advance Tubes, Becton, Dickinson and Company, Franklin Lakes, NJ, USA). To isolate plasma EDTA (PE), venous blood was collected in blood collection tubes with K3EDTA (BD Vacutainer^®^ EDTA tubes, Becton, Dickinson and Company, Franklin Lakes, NJ, USA). Serum was collected after a 10 min centrifugation at 2000x g, according to manufacturer’s instructions, whereas for PE collection a centrifugation at 500x g for 15 min was performed. All samples were stored at -80°C until further analysis (20, 26).

### 2.3 Neutrophil isolation

Peripheral blood neutrophils were isolated from heparinized blood by Histopaque double-gradient density centrifugation (11191 and 10771, Sigma-Aldrich, St Louis, MO, USA). Following the manufacturer’s instructions, whole blood was centrifuged at 700x g at 20-25°C for 30min. Isolated neutrophils were then washed once with 1x Dulbecco’s phosphate-buffered saline solution (1x PBS) (200x g, 10 min, at 20-25oC). Isolated neutrophil population purity exceeded ≥98%, as assessed by flow cytometry.

### 2.4 Human pulmonary fibroblast cell culture

Primary HPFs (Cat#: C-12360, PromoCell, Heidelberg, Germany) were cultured at 37oC with 5% CO2 in low glucose Dulbecco’s Modified Eagle Medium (DMEM, PAN Biotech, Aidenbach, Germany), supplemented with 10% v/v Fetal Bovine Serum (FBS, Capricorn Scientific, Ebsdorfergrund, Germany), 100 U/mL Antibiotics-Antimycotic solution (Biosera, Cholet, France) and 5% v/v MEM Non-Essential Amino Acids Solution (Thermo Fisher Scientific, Waltham, MA, USA). Once HPFs reached 80-85% confluency, they were sub-cultivated by using Trypsin/EDTA solution (Capricorn Scientific, Ebsdorfergrund, Germany) for cell detachment. Cells from passages 4-8 were used for this study (23).

### 2.5 Stimulation and inhibition studies

#### 2.5.1 Neutrophils

Neutrophils isolated from either patients (RA-ILD neutrophils) or healthy individuals (control neutrophils) were cultured for 3h at 37oC with 5% CO2 in Roswell Park Memorial Institute (RPMI) medium (Capricorn Scientific, Ebsdorfergrund, Germany) supplemented with 2% v/v heterologous healthy donor serum. To reproduce ex vivo observations, control neutrophils were cultured in RPMI and stimulated *in vitro* with 5% serum from patients with RA-ILD (RA-ILD serum). For all *in vitro* stimulation studies, control neutrophils were cultured for 60 min or 3h (37oC, 5% CO2), to study gene expression and NET formation, respectively. Control neutrophils that were not stimulated with RA-ILD serum served as control group in all *in vitro* experiments (20).

#### 2.5.2 HPFs

To investigate the crosstalk between neutrophils and fibroblasts, HPFs were stimulated with ex vivo-isolated NET structures (DNA concentration: 0.5 µg/ml), in DMEM at 37oC with 5% CO2. To assess the effect of NET scaffold and NET-derived interleukin (IL)-17A and tissue factor (TF) on HPFs, inhibitions were performed prior to cell stimulation. Pre-incubation of ex vivo-isolated NET structures with 1 U/mL recombinant DNase I (Takara Bio, Shiga, Japan) or 10μg/mL anti-human IL-17A antibody (R&D Systems, Minneapolis, MN, USA) was performed, to dismantle the NET scaffold and neutralize IL-17A on NETs respectively (19, 25). The effect of TF-bearing NETs was examined by protease-activated receptor-1 (PAR-1) blockade on HPFs with the FLLRN peptide (500mM, Anaspec, Fremont, CA, USA). To evaluate the simultaneous effect of both the NET scaffold and the NET-derived IL-17A and TF on HPFs, incubation with all the above inhibitors was performed at the same time. All inhibitions were performed for 30min at 37oC with 5% CO2. HPFs were cultured for 3h to study gene expression and 20h to investigate their migratory/wound healing capacity. Unstimulated HPFs were used as negative control in all *in vitro* studies (20, 21).

The concentrations and time points used to examine neutrophils and HPFs were optimized before the stimulation and inhibition studies. All substances used in these experiments were endotoxin-free, as determined by a Limulus amebocyte lysate assay (Thermo Fisher Scientific, Waltham, MA, USA).

### 2.6 NET structures generation and collection

For ex vivo NET structures generation, a total of 2 × 106 neutrophils (RA-ILD or control) were cultured in a 6-well cell culture plate (SPL Life Sciences, Kyonggi-do, Republic of Korea) in RPMI, supplemented with 2% v/v heterologous healthy donor serum, for 3h at 37oC with 5% CO2. Following, the cell culture medium was removed, and neutrophils were washed once with pre-warmed RPMI. After vigorous agitation of the culture plate and centrifugation at 20× g for 5 min, NETs were collected in the supernatant phase. Control neutrophils were used as negative control (control NETs) (20).

### 2.7 Immunofluorescence

Peripheral blood neutrophils were cultured in a 24-well cell culture plate (SPL Life Sciences, Kyonggi-do, Republic of Korea) on Poly-L-Lysine coverslips (Biocoat, NY, USA), to evaluate their capacity to release NETs and examine the protein profile of NETs. Following a 3-hour incubation (37oC, 5% CO2), neutrophils were fixed with 10% formaldehyde solution (Biognost, Zagreb, Croatia) for 30 min at 4oC. Nonspecific binding sites were blocked with 6% normal goat serum (Thermo Fisher Scientific, Waltham, MA, USA) in 1x PBS (blocking solution). The samples were stained with a primary antibody solution, consisting of an anti-human tissue factor (TF) monoclonal antibody (mAb) (1:200 dilution, BioMedica Diagnostics, Windsor, Canada), an anti-human IL-17A mAb (1:50 dilution,R&D System, Minneapolis, MN, USA) or an anti-human neutrophil elastase (NE) mAb (1:100 dilution, Abcam, Cambridge, UK) in blocking solution, for 1h at room temperature (RT). Following, incubation with a polyclonal anti-rabbit IgG AlexaFluor647 antibody (Invitrogen, Waltham, MA, USA) or a polyclonal anti-mouse IgG AlexaFluor488 antibody (Invitrogen, Walthan, MA, USA), diluted in blocking solution according to manufacturer’s instructions, was performed. Finally, cells were stained with DAPI solution (Sigma-Aldrich, St Louis, Missouri, USA) and mounted on microscope slides (Knittel Glass, Braunschweig, Germany) using a hardening mounting medium (Thermo Fisher Scientific, Waltham, MA, USA) (20, 21).

Sample visualization was performed on a Nikon ECLIPSE Ti2 Inverted Microscope (Nikon, Melville, NY, USA) with a 40× oil lens (1.30NA) and image acquisition was achieved, using NIS-Elements software (Nikon, Melville, NY, USA). Images were analyzed in Fiji software version 2.9.0 (27).

### 2.8 Citrullinated histone 3 ELISA

Concentration of CitH3 was measured in serum samples, in accordance with manufacturer’s instructions (Cayman Chemical, Ann Arbor, MI, USA).

### 2.9 Thrombin-antithrombin complex ELISA

To assess the levels of thrombin, the concentration of TAT was measured in: (a) PE from RA-ILD patients and healthy individuals and (b) ex vivo-isolated NET structures. The assay was performed based on the manufacturer’s instructions (Abcam, Cambridge, UK).

### 2.10 Interleukin-17A ELISA

IL-17A ELISA was applied to measure IL-17A concentration in: (a) serum samples and (b) *ex vivo*-isolated NET structures. The ELISA kit was utilized in accordance with manufacturer’s protocol (R&D Systems, Minneapolis, MN, USA).

### 2.11 Human terminal complement complex ELISA

Human soluble terminal complement complexes (i.e., sTCC or sC5b-9) were quantified in PE from RA-ILD patients and healthy individuals, by applying a commercially available ELISA kit in line with manufacturer’s guidelines (Hycult Biotech, Uden, The Netherlands).

### 2.12 RNA isolation, cDNA synthesis and quantitative real-time polymerase chain reaction

As formerly described, RNA isolation was performed using TRIzol reagent (Thermo Fisher Scientific, Waltham, MA, USA) according to manufacturer’s instructions. cDNA was synthesized with an appropriate kit (Takara Bio, Shiga, Japan) and RT-qPCR was conducted using a commercially available SYBR green RT-qPCR master mix (Kapa Biosystems, Wilmington, MA, USA). The expression of TF, IL-17A and RAR-related orphan receptor C (RORc) was examined in neutrophils. To evaluate the activation of HPFs, the expression of smooth muscle actin alpha 2 (ACTA2) was studied. To normalize the expression of the abovementioned genes, glyceraldehydes-3-phosphate dehydrogenase (GAPDH) was utilized, following the housekeeping gene normalization method. RT-qPCR primers were designed using Beacon Designer version 4.0. Further details regarding the primers and the RT-qPCR conditions are shown in **Supplementary Table S1**. Data analysis was performed by applying the 2-ΔΔCt method (19, 21, 28).

### 2.13 Migration/wound healing assay

To evaluate the migratory/wound healing capacity of HPFs, cells were seeded in a 24-well cell culture plate (SPL Life Sciences, Kyonggi-do, Republic of Korea). When cells reached 90% confluency, stimulation/inhibition studies were performed. Wound healing was evaluated after 20hr incubation and assessed by May-Grünwald Giemsa stain. Assay was performed following manufacturer’s instructions and recommendations (21).

### 2.14 MGG stain

MGG stain was performed to visualize the migrating/wound healing capacity of HPFs. Firstly, cells were incubated with May-Grünwald stain for 5min at RT. After washing away, the excess of May-Grünwald stain with water, a 20-minute incubation (RT) with Giemsa stain (1:10 dilution) followed. Finally, Giemsa stain was removed and HPFs were washed with water (18). Stained cells were observed under an OLYMPUS IX73 Inverted Microscope (OLYMPUS Corporation, Tokyo, Japan) with a 4x air lens (0.10NA). Images were acquired using OLYMPUS cellSens Entry software version 1.14 (OLYMPUS Corporation, Tokyo, Japan) and final images were produced with Fiji software version 2.9.0 (22).

### 2.15 Statistical analysis

Comparisons between two independent groups were performed by using the non-parametric Mann-Whitney U test (two-tailed), whereas the statistical analysis between two paired groups was achieved by applying the non-parametric Wilcoxon matched-pairs signed rank test (two-tailed). Statistical comparisons between three paired groups were performed via the Friedman test. Spearman’s rank correlation coefficients test at 95% confidence intervals (CI) was utilized for bivariate correlation analysis. Data in all graphs are presented as Mean ± standard deviation (SD) and the cut-off value for statistical significance was set to P < 0.05. Statistical analysis of the experimental data was conducted with the use of GraphPad Prism version 8 (GraphPad Software, Inc., San Diego, CA, USA).

## 3 Results

### 3.1 Inflammatory mediators are found in the circulation of patients with RA-ILD

**Figure 1:**
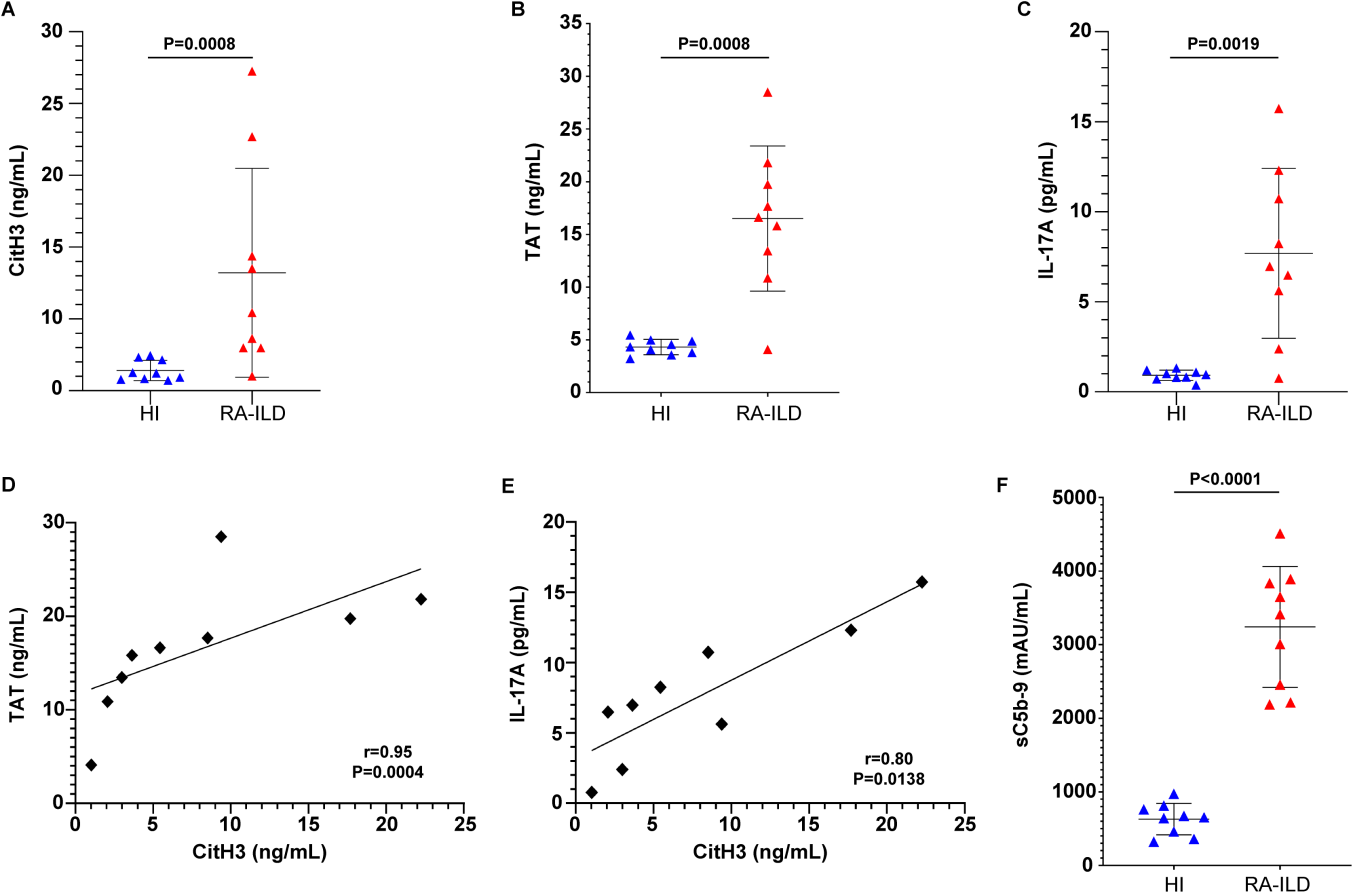
Inflammatory markers are detected in the circulation of patients with rheumatoid arthritis-interstitial lung disease (RA-ILD). Levels of **(A)** CitH3 representing NET release, **(B)** thrombin anti-thrombin (TAT) complex and **(C)** interleukin (IL)-17A in the serum or plasma of patients with RA-ILD, compared to healthy individuals (HI) (*n* = 9 subjects per group). Correlation between **(D)** TAT and CitH3 levels or **(E)** IL-17A and CitH3 levels. Spearman’s r and P values are shown. Levels of **(F)** soluble terminal complement complex sC5b-9 in the plasma of patients with RA-ILD, compared to healthy individuals (HI) (*n* = 9 subjects per group). For **(A, B, C, F)**, data are shown as mean ± SD, Mann-Whitney U test (two-tailed). All conditions were compared to HI. Statistically significant: P < 0.05.

Inflammatory mediators were assessed in serum samples collected from patients diagnosed with RA-ILD and healthy controls (**Table 1**). Using protein immunoassays, we observed elevated levels of circulating NETs, measured by CitH3 ELISA, in active RA-ILD patients compared to controls (**Figure 1A**). Additionally, RA-ILD sera exhibited increased TAT activity (**Figure 1B**) and IL-17A expression (**Figure 1C**). Importantly, these inflammatory proteins correlated positively with NET levels (**Figures 1D, E**). Moreover, RA-ILD patients showed significantly elevated plasma levels of sC5b-9, detected by TCC ELISA (**Figure 1F**), underscoring a pro-inflammatory microenvironment conducive to disease progression.

### 3.2 RA-ILD patients release neutrophil extracellular traps enriched with tissue factor and interleukin-17A

**Figure 2:**
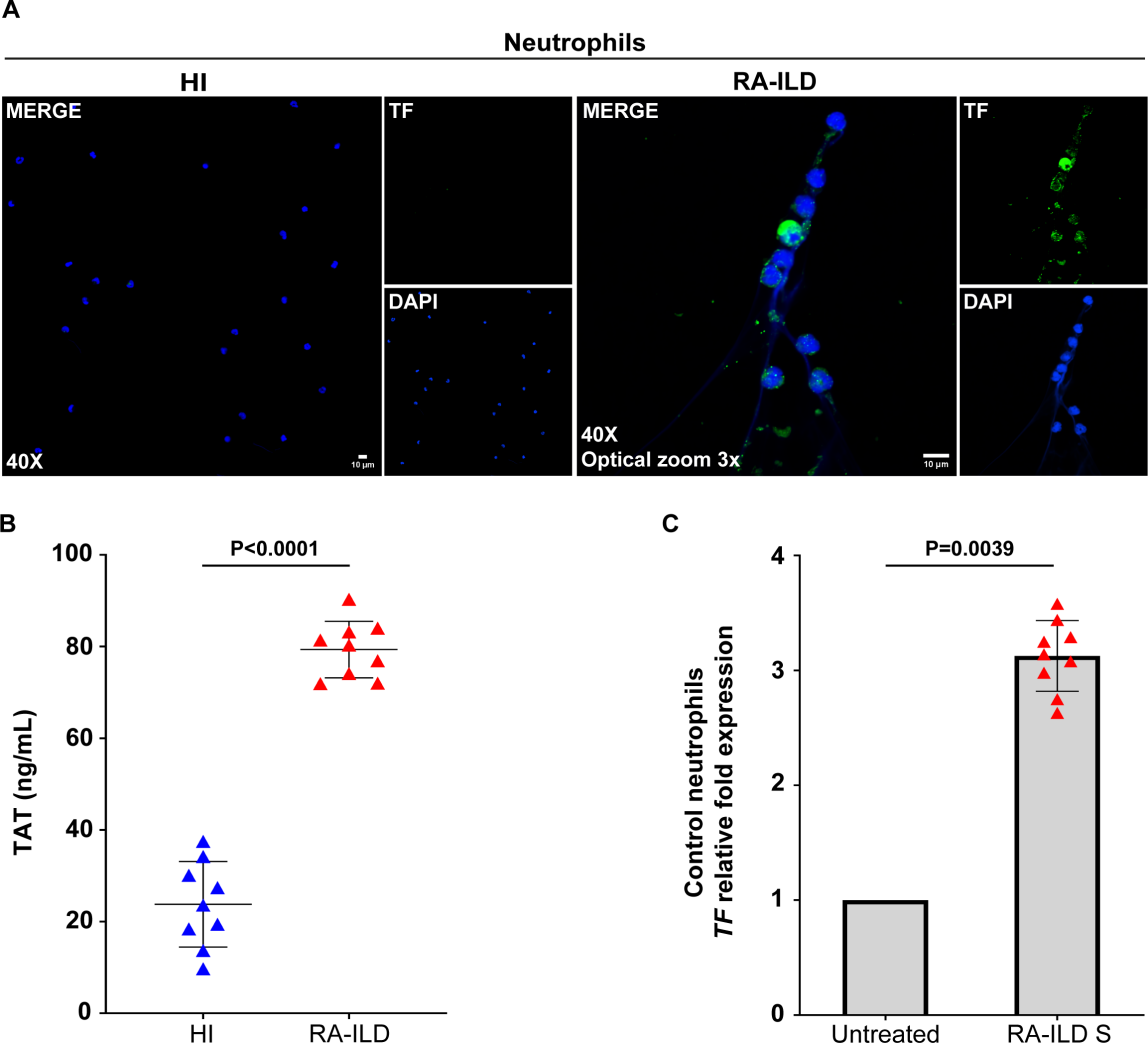
Neutrophils isolated from rheumatoid arthritis-interstitial lung disease (RA-ILD) patients release neutrophil extracellular traps (NETs) expressing tissue factor (TF). **(A)** Fluorescence microscopy images showing TF staining (blue, DAPI; green, TF; original magnification, 400×; optical zoom, 3x) in neutrophils collected from RA-ILD patients. A representative example of 9 independent experiments is shown. **(B)** Thrombin anti-thrombin (TAT) levels in *ex vivo*-isolated NET structures from RA-ILD patients, compared to healthy individuals (HI) (*n* = 9 subjects per group). **(C)** *TF* mRNA expression in control neutrophils treated with RA-ILD serum (RA-ILD S), as assessed by real-time qPCR (*n* = 9 subjects per group). For **(B)** data are shown as mean ± SD, Mann-Whitney U test (two-tailed). For **(C)** data are shown as mean ± SD, Wilcoxon matched-pairs signed rank test (two-tailed). All conditions were compared to HI/Untreated. Statistically significant: P < 0.05.

Further characterization of neutrophils from active RA-ILD patients revealed spontaneous release of NETs enriched with functional TF, as assessed by confocal microscopy (**Figure 2A**) and TAT assay in ex vivo-isolated NET structures (**Figure 2B**). Similarly, *in vitro* experiments demonstrated that RA-ILD serum induced TF mRNA expression in control neutrophils (**Figure 2C**).

**Figure 3:**
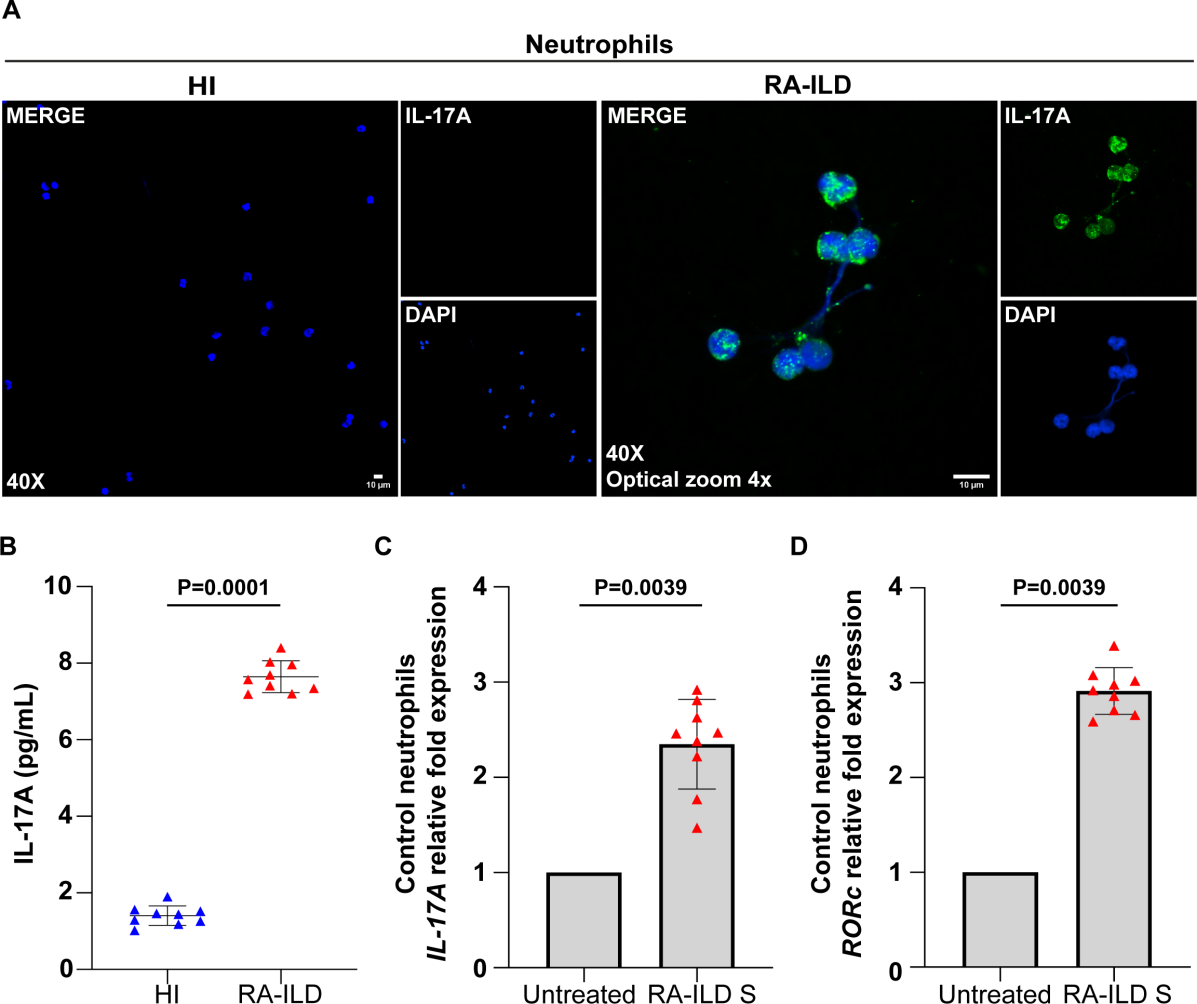
Neutrophils collected from rheumatoid arthritis-interstitial lung disease (RA-ILD) patients release neutrophil extracellular traps (NETs) expressing interleukin (IL)-17A. **(A)** Fluorescence microscopy images showing IL-17A staining (blue, DAPI; green, IL-17A; original magnification, 400×; optical zoom, 4x) in neutrophils isolated from RA-ILD patients. A representative example of 9 independent experiments is shown. **(B)** IL-17A levels in *ex vivo*-isolated NET structures from RA-ILD patients, compared to healthy individuals (HI) (*n* = 9 subjects per group). **(C)** *IL-17A* and **(D)** *RAR-related orphan receptor C* (*RORc*) mRNA expression in control neutrophils treated with RA-ILD serum (RA-ILD S), as assessed by real-time qPCR (*n* = 9 subjects per group). For **(B)** data are shown as mean ± SD, Mann-Whitney U test (two-tailed). For **(C, D)** data are shown as mean ± SD, Wilcoxon matched-pairs signed rank test (two-tailed). All conditions were compared to HI/Untreated. Statistically significant: P < 0.05.

Additionally, NETs from RA-ILD patients were found to be coated with IL-17A, confirmed by immunofluorescence (**Figure 3A**) and IL-17A NET ELISA (**Figure 3B**). Control neutrophils stimulated with RA-ILD serum showed intracellular overexpression of IL-17A and RORc, a transcription factor associated with IL-17 regulation (**Figures 3C, D**). These findings collectively highlight the increased formation of TF- and IL-17A- bearing NETs in active RA-ILD, implicating neutrophils in the induction of disease-associated inflammation.

### 3.3 TF and IL-17A-bearing neutrophil extracellular traps enhance the fibrotic potential of pulmonary fibroblasts *in vitro*

**Figure 4:**
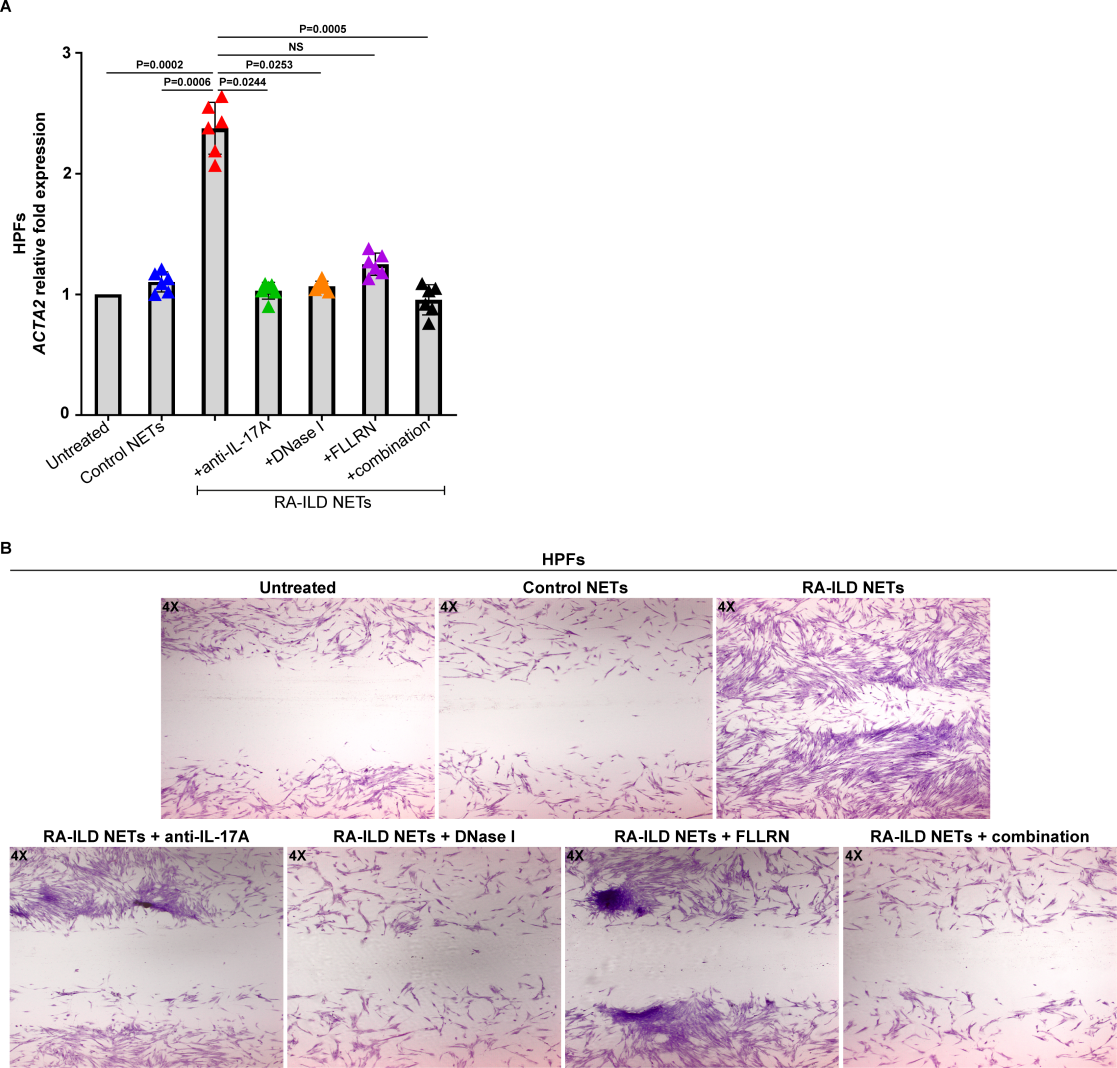
Human pulmonary fibroblasts (HPFs) acquire a dynamic phenotype upon co-culture with NETs from rheumatoid arthritis-interstitial lung disease (RA-ILD) patients. **(A)** *Smooth muscle actin alpha 2* (*ACTA2*) mRNA expression in HPFs treated with inflammatory NETs released from RA-ILD patients (RA-ILD NETs), as assessed by real-time qPCR (*n* = 6 independent experiments). Data are shown as mean ± SD, Friedman test. All conditions were compared to Untreated. Simultaneous inhibition with anti-IL-17A, DNase I and FLLRN is mentioned as combination. Statistically significant: P < 0.05; NS: not significant. **(B)** Migration/wound healing potential of HPFs stimulated with RA-ILD NETs, as assessed by light microscopy (May-Grünwald Giemsa staining, original magnification, 40×). A representative example of 6 independent experiments is shown. RA-ILD NETs were pre-incubated with DNase I or a neutralizing antibody against human IL-17A, to dismantle NETs or hinder IL-17A signaling, respectively. To block thrombin signaling, HPFs were pre-incubated with the FLLRN peptide.

To understand the impact of inflammatory NETs on tissue-resident cells, HPFs were incubated with NET structures released by active RA-ILD patients (RA-ILD NETs). RA-ILD NETs triggered the activation of HPFs, as evidenced by the up-regulation of ACTA2 (**Figure 4A**). In addition, after this stimulation, HPFs showed increased proliferation/migration rates (**Figure 4B**).

On the other hand, disassembly of RA-ILD NETs with DNase I abolished the fibrotic potential of HPFs (**Figures 4A, B**), suggesting a specific effect of NETs on the fibrotic process. In addition, protein components of RA-ILD NETs, namely TF and IL-17A, also exert a direct effect on the fibrotic activity of HPFs. Particularly, the pretreatment of cells with the FLLRN peptide, which blocks thrombin signaling, resulted in a significant reduction of HPFs fibrotic potential (**Figures 4A, B**). The neutralization of IL-17A on RA-ILD NETs with a monoclonal antibody also led to a similar result (**Figures 4A, B**). Consequently, these observations indicate RA-ILD NETs as potent mediators of tissue damage and suggest multiple targets for therapeutic interventions in RA-ILD.

### 3.4 Administration of nintedanib in RA-ILD patients moderates the release of neutrophil extracellular traps and sC5b-9; a small-scale preliminary study

**Figure 5:**
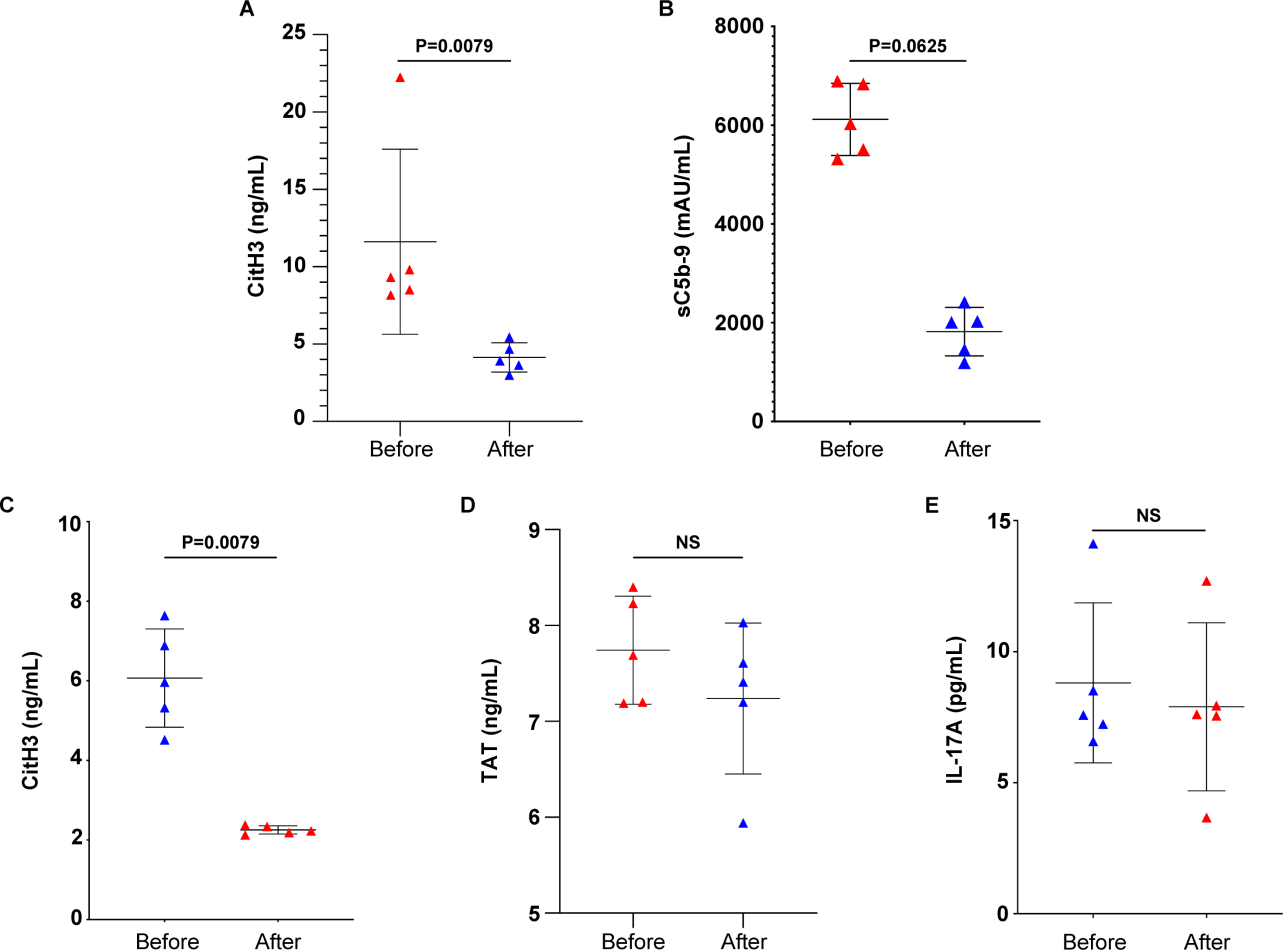
Nintedanib reduces NET formation and complement activation in rheumatoid arthritis-interstitial lung disease (RA-ILD) patients. Levels of **(A)** CitH3 indicating NET release and **(B)** soluble terminal complement complex sC5b-9 in the serum or plasma of patients with RA-ILD, before and 16 weeks after the initiation of the antifibrotic therapy (*n* = 5 subjects per group). Levels of **(C)** CitH3, **(D)** thrombin anti-thrombin (TAT) complex and **(E)** interleukin (IL)-17A in *ex-vivo* isolated NET structures from patients with RA-ILD, before and 16 weeks after the initiation of the antifibrotic therapy (*n* = 5 subjects per group). Data are shown as mean ± SD, Wilcoxon matched-pairs signed rank test (two-tailed). Statistically significant: P < 0.05; NS, not significant.

Prompted by recent studies discussing the effects of novel antifibrotic agents on the progression of RA-ILD (10), we next performed a paired analysis in samples derived from five RA-ILD patients, with samples taken before and 16 weeks after starting treatment with nintedanib (**Table 1**). Blood serum obtained from patients with RA-ILD under treatment with nintedanib (treated patients) showed a decrease in circulating CitH3 compared to the same patients before the initiation of the antifibrotic therapy, as verified by ELISA immunoassay (**Figure 5A**). In addition, treated RA-ILD patients were characterized by reduced levels of sC5b-9 in plasma, as evidenced by TCC ELISA assay (**Figure 5B**).

The effect of nintedanib was also assessed on ex vivo-isolated NET structures. RA-ILD-treated patients showed lowered NET formation compared to the same patients before the initiation of therapy, as evidenced by CitH3 ELISA (**Figure 5C**). In contrast, the presence of TF (**Figure 5D**) and IL-17A (**Figure 5E**) in ex vivo-isolated NETs was not found to be reduced, as indicated by ELISA assays. This observation was further verified by mRNA studies (**Supplementary Figure S1**). Together, these observations support that nintedanib could have a regulatory effect on NET formation and sC5b-9 release in patients with RA-ILD.

## 4 Discussion

Though recent data have identified RA-ILD as the second leading cause of death in RA patients (29, 30), the molecular mechanisms underlying the development and progression of the disease remain poorly understood. Our study offers new evidence supporting the involvement of neutrophils in the progression of RA-ILD, through disease-specific inflammatory NETs.

It is well-documented that the role of neutrophils in RA pathophysiology is multifactorial (31). Neutrophils can form NETs, which are induced by the autoantibody complexes and coated with citrullinated autoantigens (32). The formation of NETs is a citrullination-dependent process and, as previous evidence suggests, the citrullination pathway is upregulated in bronchoalveolar lavage (BAL) cells of RA-ILD patients (33). Additionally, other NET-associated mechanisms, such as autophagy and oxidative stress, are also elevated in RA-ILD conditions, contributing significantly to the course of the disease (8). In accordance with the above data, our results indicate NETs as important contributors in the RA-ILD pathophysiology.

More specifically, neutrophils can play a prominent role in the vicious cycles of interstitial lung inflammation and fibrosis. They can influence the progression of ILD via promoting ECM accumulation and fibroblast activation (34). In parallel, the neutrophil-to-lymphocyte ratio (NLR) in the blood is correlated with the possibility of ILD occurrence and the outcome of the disease (35). Our data suggest that inflammatory NETs can interact with HPFs, which subsequently escape the quiescent state and become significantly activated. On the contrary, upon disruption of NETs integrity with DNase I, the activation level of HPFs is reduced. Consequently, our study proposes that neutrophils/NETs could be involved in RA-ILD exacerbations and pulmonary fibrosis progression.

Several cytokines are implicated in RA-ILD pathogenesis, promoting inflammatory responses and fibrogenesis (7, 36, 37). Notably, a proteomic analysis – conducted in serum samples from RA-ILD patients – shows a marked increase in IL-17A, compared to RA-noILD group of patients (38). Although IL-17A is considered as the signature cytokine of a subset of CD4+ helper T cells (Th17), it can also be expressed by activated neutrophils/NETs (19, 21, 25, 39). Indeed, our data suggest that neutrophils/NETs express IL-17A and, hence, they can act as an alternative source of IL-17A in RA-ILD.

Additionally, previous studies have linked IL-17A to fibrotic responses in RA-ILD. Particularly, a research - examined lung biopsies from RA-ILD patients, supported that pathogenic fibroblasts overexpress IL-17A receptor (IL-17RA), in contrast to cells from either normal or idiopathic pulmonary fibrosis lung tissue (24). Our approaches indicate that IL-17A-bearing NETs can interact with HPFs, enhancing their activation and migration capacity.

Moreover, it has been cited that plasma and synovial fluid of patients with RA can be characterized by high TF activity and elevated levels of coagulation factor VIII (40). Beyond its role in thrombosis, TF/thrombin axis can also contribute to fibroblast proliferation (41), wound healing (42), and inflammation (20). Markedly, thrombin can drive inflammatory responses acting through PAR-1 receptor (43). Indeed, this receptor is up-regulated in systemic sclerosis-associated ILD patients (44). In line with current knowledge, our evidence demonstrates that RA-ILD NETs are coated with bioactive TF and increase the fibrotic dynamic of HPFs.

The involvement of complement system in RA has been extensively studied. Research has shown that there are decreased levels of C3 and C4 proteins and higher levels of complement activation products such as C3a, C5a, and soluble C5b-9 (sC5b-9) in the synovial fluid of RA patients (45, 46). In addition, elevated sC5b-9 levels in RA plasma suggest that complement activation extends beyond joint inflammation, impacting other organs and tissues (9). Moreover, complement factors, along with their receptors, have been described in both acute and chronic lung pathologies due to their involvement in thrombo-inflammatory events or/and tissue injury (20, 47, 48). Here, we observe a significant elevation of sC5b-9 in plasma samples from patients with active RA-ILD compared to healthy individuals, indicating a potential association with disease activity. A correlation between CitH3 and sC5b-9 in RA-ILD patients was also detected, underscoring the evident interplay between the complement cascade activation and NETs.

To date, managing the complexities of RA-ILD remains a challenge and, even though various therapeutic strategies are proposed, comprehensive randomized controlled trials specific to RA-ILD are lacking (7, 8, 49). Clinical trials investigating the blockade of IL-17A have been conducted in RA patients, demonstrating modest efficacy compared to its effects in psoriasis, psoriatic arthritis, and spondylarthritis (50). Notably, the limited success of IL-17A blockade in RA may be attributed to several plausible reasons. Experimental arthritis models suggest that IL-17A-producing cells may play a more critical role in the erosive stages of RA rather than early disease onset (51, 52), indicating potential benefits of IL-17A blockade in specific subsets of RA patients.

Nintedanib, an antifibrotic agent, has been shown to prevent the progression of RA-ILD by targeting a range of kinases, including platelet-derived growth factor (PDGF) receptors α and β, various vascular endothelial growth factor (VEGF) receptor subtypes, and fibroblast growth factor (FGF) receptor types 1, 2, and 3 (13, 53). PDGFs play a crucial role in promoting inflammation; they enhance neutrophil migration to the sites of platelet (PLT) release, potentially facilitating interaction between PLTs and neutrophils, leading to NETosis (54, 55). PDGFs can also activate the complement system, through the classical and alternative pathway (56). Studies using bleomycin-induced pulmonary fibrosis models have demonstrated nintedanib’s ability to mitigate neutrophil chemotaxis (57) as well as reduce neutrophils and lymphocytes in BAL fluid (58). Our findings support the notion that nintedanib administration may effectively regulate complement activation and NET formation in patients with RA-ILD.

Our study has provided valuable insights, but some limitations need to be acknowledged. Firstly, the small sample size, with only nine patients and nine healthy individuals, suggests that our findings may not be broadly applicable and may not fully represent the wider RA-ILD population. Secondly, because the study is cross-sectional, we cannot establish a cause-and-effect relationship between NET formation and disease progression. Additionally, relying on specific biomarkers, such as CitH3 and IL-17A, may not capture the full range of inflammatory and fibrotic processes involved in RA-ILD. Finally, the short duration of the nintedanib treatment assessment (16 weeks) restricts our understanding of the long-term therapeutic benefits and potential side effects. This necessitates further longitudinal studies to confirm our initial observations.

## 5 Conclusions

Collectively, our study supports that peripheral blood neutrophils can play a key role in the immunofibrotic aspects of RA-ILD. The disease microenvironment enhances the release of inflammatory NETs, which can subsequently increase the fibrotic dynamic of human pulmonary fibroblasts. The formation of NETs is positively correlated with complement activation, whereas the administration of nintedanib reduces both complement activation and NETs, offering a promising avenue for disease management. Further studies will provide a better understanding of the complex molecular mechanisms involved in RA-ILD and tailor therapeutic approaches according to the individual patient profile.

## Data Availability

The raw data supporting the conclusions of this article will be made available by the authors, without undue reservation.

## Supplementary material

Supplementary Figure 1
*TF*, *IL-17A* and *RORc* levels are not reduced in rheumatoid arthritis-interstitial lung disease (RA-ILD) patients before and after nintedanib. mRNA levels of **(A)** *TF*, **(B)** *IL-*17A and **(C)** *RORc* in healthy individual neutrophils after *in vitro* stimulations with serum from patients with RA-ILD, before and 16 weeks after the initiation of the antifibrotic therapy (*n* = 5 subjects per group). For **(A–C)**, data are shown as mean ± SD, Wilcoxon matched-pairs signed rank test (two-tailed). Statistically significant: P < 0.05; NS: not significant.
