# Supplementary figures and images for: Neutrophil extracellular traps as immunofibrotic mediators in RA-ILD; pilot evaluation of the nintedanib therapy
Source: Front Immunol. 2024 Oct 23;15:1480594. doi: 10.3389/fimmu.2024.1480594 (PMC11538023; doi:10.3389/fimmu.2024.1480594)

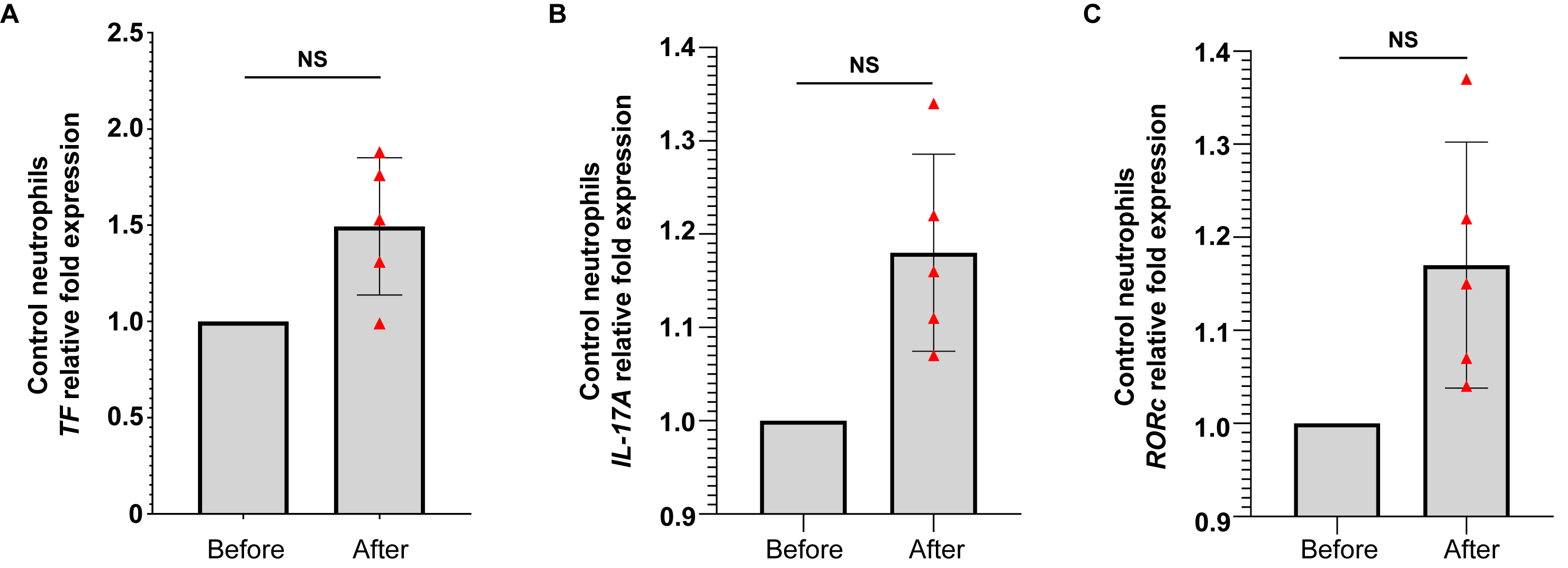

Supplement: Supplementary Figure 1 — TF, IL-17A and RORc levels are not reduced in rheumatoid arthritis-interstitial lung disease (RA-ILD) patients before and after nintedanib. mRNA levels of (A) TF, (B) IL-17A and (C) RORc in healthy individual neutrophils after in vitro stimulations with serum from patients with RA-ILD, before and 16 weeks after the initiation of the antifibrotic therapy (n = 5 subjects per group). For (A–C), data are shown as mean ± SD, Wilcoxon matched-pairs signed rank test (two-tailed). Statistically significant: P < 0.05; NS: not significant. [file Image1.tif]
