# Supplementary material for: Neutrophil extracellular traps as immunofibrotic mediators in RA-ILD; pilot evaluation of the nintedanib therapy
Source: Front Immunol. 2024 Oct 23;15:1480594. doi: 10.3389/fimmu.2024.1480594 (PMC11538023; doi:10.3389/fimmu.2024.1480594)
Supplement: Supplementary file 2 [file Table1.docx]

| **Supplementary Table 1.** Sequence of primers and RT-qPCR conditions | | | |
| --- | --- | --- | --- |
| **Gene** | **Primer** | **Primer Sequence** | **RT-qPCR conditions** |
| *RORc* | FRD | 5’ GTGGGGACAAGTCGTCTGG 3’ | 1. 52^o^C for 5 min  2. 95 ^o^C for 2 min  3. 35 cycles of:  I. 95 ^o^C for 15 sec  II. 56^o^C for 40 sec  4. 52 ^o^C for 5 min  5. Melting curve analysis |
|  | REV | 5’ AGTGCTGGCATCGGTTTCG 3’ |  |
| *TF* | FRD | 5’ AACCCGTCAATCAAGTCTACAC 3’ |  |
|  | REV | 5’ TCACATCCTTCACAATCTCGTC 3’ |  |
| *ACTA2* | FRD | 5’ ACGCACAACTGGCATCG 3’ |  |
|  | REV | 5’ CGGACAATCTCACGCTCAG 3’ |  |
| *GAPDH* | FRD | 5’ AGGTGGTCTCCTCTGACTTC 3’ |  |
|  | REV | 5’ CTGTTGCTGTAGCCAAATTCG 3’ |  |
| *IL-17A* | FRD | 5’ TGGTGTCACTGCTACTG 3’ | 1. 52 ^o^C for 5 min  2. 95 ^o^C for 2 min  3. 35 cycles of:  I. 95 ^o^C for 15 sec  II. 51^o^C for 40 sec  4. 52^o^C for 5 min  5. Melting curve analysis |
|  | REV | 5’ CATTGCGGTGGAGATTC 3’ |  |
| *GAPDH* | FRD | 5’ GGGAAGCTTGTCATCAATGG 3’ |  |
|  | REV | 5’ CATCGCCCCACTTGATTTTG 3’ |  |
| FRD: forward primer, REV: reverse primer | | | |
